# Metal Material Processing Using Femtosecond Lasers: Theories, Principles, and Applications

**DOI:** 10.3390/ma17143386

**Published:** 2024-07-09

**Authors:** Zhicong He, Lixiang Lei, Shaojiang Lin, Shaoan Tian, Weilan Tian, Zaiyuan Yu, Fang Li

**Affiliations:** 1 School of Mechanical and Electrical Engineering, Hubei Key Laboratory of Intelligent Transportation Technology and Device, Hubei Polytechnic University, Huangshi 435003, China; hzc_900503987@163.com (Z.H.); yuzy0630@163.com (Z.Y.); 2 School of Mechanical and Electrical Engineering, Hubei Key Laboratory of Optical Information and Pattern Recognition, Wuhan Institute of Technology, Wuhan 430073, China; 3 Hubei Zhongpei Electronic Technology Limited Company, Huangshi 435200, China; 4 Hubei Chuangjie Biotechnology Technology Limited Company, Huangshi 435200, China

**Keywords:** femtosecond laser, surface modification, ablation thresholds, liquid metal processing

## Abstract

Metal material processing using femtosecond lasers is a useful technique, and it has been widely employed in many applications including laser microfabrication, laser surgery, and micromachining. The basic mechanisms of metal processing using femtosecond lasers are reviewed in this paper and the characteristics and theory of laser processing are considered. In addition to well-known processes, the recent progress relating to metals processing with femtosecond lasers, including metal material drilling, metal ablation thresholds, micro/nano-surface modification, printed circuit board (PCB) micromachining, and liquid metal (LM) processing using femtosecond lasers, is described in detail. Meanwhile, the application of femtosecond laser technology in different fields is also briefly discussed. This review concludes by highlighting the current challenges and presenting a forward-looking perspective on the future of the metal laser processing field.

## 1. Introduction

As one of the most significant achievements in the 20th century, laser technology has made outstanding contributions to the development of various industries [1,2,3,4,5]. The application fields of laser technology continue to expand, including agriculture [6,7,8], industry [9,10,11], national defense [12,13,14], and scientific research [15,16,17]. It has gradually become an indispensable key technology.

The principle of the laser was anticipated as early as 1916 based on Einstein’s stimulated radiation theory [18]. Theodore Maiman successfully developed the world’s first operational laser in 1960, utilizing ruby as a gain medium with a wavelength of 694.3 nm [19]. In 1965, picosecond pulse generation was achieved, marking the advent of ultrashort pulse times [20]. In the 1980s, the pulse width of collisional mode-locked ring dye lasers reached 27 fs, which was shortened to 6 fs after compensating for dispersion by grating and prism pairs [21]. The peak power reached the 10^15^ W level, which promoted the development of femtosecond laser technology. However, obvious disadvantages include the inconvenient treatment and maintenance of dye solutions, as well as the poor repeatability and operation stability. In the 1990s, a breakthrough of great significance was made during the study of titanium-doped sapphire laser technology: due to sapphire’s fluorescence spectrum bandwidth of about 400 nm, which reaches the limitation of the pulse width at ~2.7 fs, perfect physical and optical properties are clearly observed in these titanium sapphire crystals [22,23,24,25].

Recently, ultrafast laser micro and nano manufacturing has become one of the frontiers in the development of processing technology [26,27,28]. It is worth noting that the remarkable decrease in the pulse width and the significant enhancement in peak power render femtosecond lasers even more advantageous in the realm of micromachining and manufacturing. Moreover, the interaction between femtosecond lasers and materials exhibits exceptional resolution and accuracy, perfectly aligning with the requirements for nanoscale processing [29,30,31]. Additionally, both the internal heat and diffusion range are not obvious in micromachining processing using femtosecond lasers, which can be regarded as “cold” fabrication.

A femtosecond laser, with its extremely short time scale (~10^−15^ s) and high energy density (>10^14^ W/cm^2^), can be precisely focused to the nanometer scale (~10^−9^ m) [32,33,34,35]. Under extreme conditions, it will process almost any material with high-quality and precision. Furthermore, laser technology has made great contributions to human society, having been conceived more than half a century ago. With the comprehensive application of lasers, many fields have made great progress, including the fields of micro/nano-devices [36], biotechnology [37], medical diagnostic systems [38], and 3D complex structure machining [39]. Hence, the regulation of laser–electron interactions or electron dynamics is crucial for advancing the field of femtosecond lasers, and it also presents novel challenges for measuring and controlling electron behavior in manufacturing processes. Consequently, theory development and observation systems must be synchronized with advancements in laser processing methods and applications.

Here, we summarize the recent advances in metal material processing using femtosecond lasers. Firstly, the development of lasers, especially femtosecond lasers, is introduced, including the history, advantages, and typical properties of femtosecond lasers. The characteristics of laser processing with different pulse widths (long pulses and femtosecond pulse widths) are also discussed. Furthermore, the progress related to metal processing using femtosecond lasers within the last 5 years is also described, such as with metal material drilling, metal ablation thresholds, micro/nano-surface modification, PCB micromachining, and LM processing. Challenges and perspectives in metal material processing with femtosecond lasers are presented.

## 2. Theory of Metal Material Processing Using Femtosecond Lasers

### 2.1. Characteristics of Laser Processing

**Figure 1:**
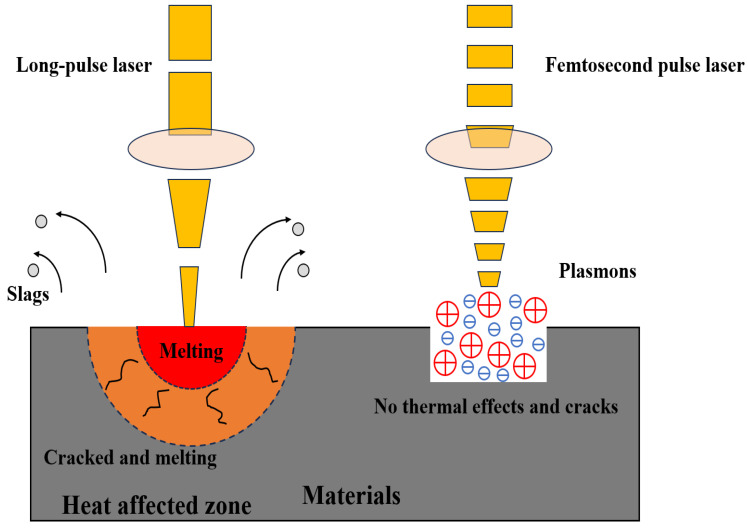
The interaction processes of the long-pulse laser (**left**)/femtosecond laser (**right**) and materials.

The interaction between materials and femtosecond lasers is an ultrafast nonlinear and nonequilibrium process [40,41,42]. On a timescale, laser processing with pulse widths is greater than the femtosecond scale: in long-pulse-width laser processing, the laser pulse is longer than the thermal diffusion time, and the heat energy continues to diffuse inside and around materials, which makes the material in the processing area remelt and the thermal influence range expand, resulting in a poor processing quality. The processing materials using long-pulse-width lasers can be seen in Figure 1 (left). It cannot be ignored that the presence of thermal diffusion leads to the following problems:

(1) Heat loss in the processing area will reduce the energy efficiency and processing utilization rate.
(2) A large amount of heat diffusion will make it difficult for the processing area temperature to reach the material’s melting point. Under a continuous pulse energy, materials are transformed from a solid state to a liquid state repeatedly, resulting in slags, which are similar to volcanic vents, thus reducing the processing quality.
(3) The existence of thermal diffusion will expand the processing area, and it will be difficult to achieve micro-fine precision machining.
(4) The presence of heat will lead to thermal expansion and contraction, and diffusion will affect mechanical stress, causing the surrounding materials near the heat affected zone (HAZ) to inevitably crack and melt.

In femtosecond laser processing, the materials can be highly ionized and expelled from the processing area as plasmons, which is due to both the short pulse width and high peak power. Simultaneously, ablation-induced heat can be efficiently dissipated, thereby circumventing thermal-diffusion-related issues. The processing of materials using femtosecond lasers can be seen in Figure 1 (right). It is worth paying close attention to the following advantages of femtosecond laser processing:

(1) A weak thermal effect: When a femtosecond laser interacts with a metal surface, it causes rapid heating of electrons on a femtosecond timescale due to their low specific heat capacity. Consequently, the surface material undergoes instantaneous ionization and ejection from inside, carrying heat energy away and reducing the temperature. This entire process is significantly shorter than lattice heat conduction. No thermal effects and cracks occur in materials.
(2) Wide applicability: The pulse duration is on a femtosecond scale, with a high power density, and the nonlinear absorption effect plays a major role in the ablation procedure, achieving excellent micro/nano-processing in diamonds, silicon, ceramics and other materials.
(3) Submicron machining accuracy: As a Gaussian beam, the typical energy distribution of a femtosecond laser is high energy in the spot’s center and low energy at the edge. Ablation only occurs when the laser energy density is higher than the threshold and the processing accuracy is less than the diffractive limitation, thus extending it to the submicron level.
(4) An accurate ablation threshold: Only when the laser energy accumulates to a certain degree will ablation occur. Once the laser’s pulse width is fixed, the materials are ablated while the laser energy density exceeds the threshold. Nonlinear absorption plays a leading role in ultrashort-pulse-width laser processing, and the ablation threshold deviation of ultrashort-pulse lasers can be ignored, resulting in clear ablation.

### 2.2. Theory of Metal Material Processing Using Femtosecond Lasers

A great number of free electrons are aggregated in metal materials. When a metal material interacts with low-energy short-pulse lasers, due to both the inverse absorption and small specific heat property of electrons, they will first absorb the photon energy, then heat transfer occurs between coupled electrons and lattices, and a thermal equilibrium occurs on the picosecond scale. The macroscopic continuous Fourier heat-conduction model holds that the electrons and the lattice are always in equilibrium. According to the above analysis, this model is not suitable for describing the interaction processes between ultrashort-pulse lasers and materials. In 1974, a two-temperature model was proposed by a Soviet scholar [43], Anisimov S., which comprehensively considers two different interaction systems, namely, photon–electron and electron–lattice models, when an ultrashort pulse acts on a metal target. Furthermore, a differential equation describing the temperature of the electron and lattice under the action of a femtosecond laser was obtained:

(1) $$C_{e}\frac{\partial T_{e}}{\partial t}=\frac{\partial }{\partial x}(k_{e}\frac{\partial T}{\partial x})-G(T_{e}-T_{l})+S(x,t)$$

(2) $$C_{l}\frac{\partial T_{l}}{\partial t}=G(T_{e}-T_{l})$$

where the subscripts *e* and *l* represent the parameters of the electron and lattice, *t* is the time, *x* is the depth, *C* represents the heat capacities (per unit volume), *T* is the temperature, *G* is the electron–lattice coupling coefficient, and *S* represents the heating source. Furthermore, S = I(t)Aαexp(−αx), where α is the material’s absorption coefficient; R represents the metal surface reflectance rate; and A = 1 − R, which is the surface transmissivity.

**Figure 2:**
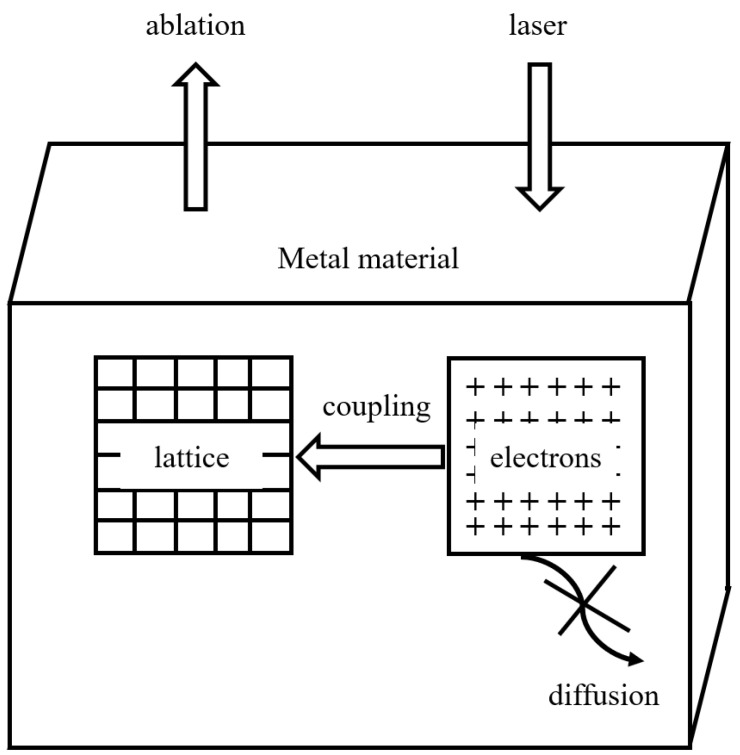
The interaction between ultrashort pulse lasers and the electron lattice. Adapted from Ref. [44].

In this two-temperature system, the femtosecond pulse ablates the metal to achieve material eruption, and this can be regarded as a direct solid plasmon or solid gas conversion; thus, the electronic and lattice parts can be analyzed as two independent sub-systems [44]. The interactions between ultrashort-pulse lasers and the electron and lattice are shown in Figure 2.

During the interaction between a femtosecond and a material, the heat capacity of the electron is smaller than that of the lattice [45,46,47,48]. The electron firstly absorbs the photon energy to heat up rapidly, and then the lattice absorbs the energy to achieve a rising temperature through the coupling effect [49]. The electron–phonon scattering process is relatively slow, generally tens to hundreds of picoseconds, and the temperature rises as a result of the lattice absorbing energy on the picosecond time scale. Once the lattice temperature reaches the metal phase transition explosion temperature, ablation damage occurs, removing material via eruption. The thermal diffusion time is very short (often negligible) in this whole cycle, so processing using femtosecond laser usually has no thermal effects and does not cause cracks.

## 3. Recent Progress in Metal Processing Using Femtosecond Lasers

The essence of laser micromachining is as follows: the laser transmits energy to an object, causing the material to melt, evaporate, and undergo physical or chemical changes for processing [50]. Several laser technologies exist, including ultraviolet lasers [51], nanosecond lasers [52], picosecond lasers [53], and femtosecond lasers [54]. Femtosecond laser processing comprises the interaction between an ultrashort pulse of a focused beam with a super-strong power, and a material, which repairs, adjusts, or removes target materials. Femtosecond lasers demonstrate great advantages in terms of processing precision and selective features, expanding the field of metal laser processing and thus contributing to laser development.

### 3.1. Metal Material Drilling Using Femtosecond Lasers

For some refractory metals, such as molybdenum, tantalum, rhenium, tungsten, and nickel, the melting points are in the range of 2610 to 3410 °C. Thus, it is difficult to achieve delicate processing using traditional long-pulse lasers. However, femtosecond lasers, which are based on multi-photon absorption and ionization mechanisms, avoid heat conduction effects, and they have gradually become the preferred choice for the high-precision treatment of these metals.

**Figure 3:**
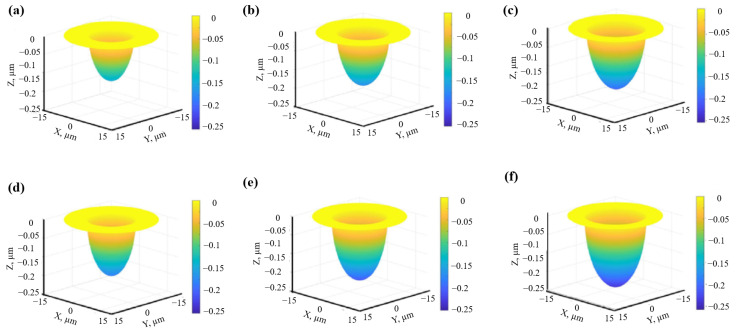
(**a**–**c**) Crater morphologies and (**d**–**f**) melting front contour profiles for various single-pulse energies of femtosecond laser: (**a**,**d**) 20 μJ; (**b**,**e**) 120 μJ; (**c**,**f**) 720 μJ [55].

Yang et al. [55] investigated laser irradiation on the surface of Ni-based and Fe-based superalloys through 3D simulation. In their study, when the single-pulse energy increased from 20 μJ to 120 μJ and 720 μJ, the depth and melting front diameter changed only a little, which contradicts the view that the machining efficiency is mainly determined by the pulse energy. Figure 3a–c display the crater morphology of various single-pulse energies in the thermodynamic equilibrium state, and the corresponding recast layer/matrix interface contour profiles.

Xu et al. [56] conducted an experiment (with a central wavelength of ~800 nm, a pulse duration of ~30 fs, and a repetition rate of ~110 Hz) to determine the single-pulse laser ablation threshold and ablation rate of Ti alloy (Ti-6Al-4V) and Al alloy (Al7075). The results showed that the ablation threshold of Ti alloy was 0.29 J/cm^2^, and that of Al alloy was 0.61 J/cm^2^. In addition, it was proposed that the incubation behavior was due to the accumulation of plastic deformation resulting from laser-induced thermal stress fields.

**Figure 4:**
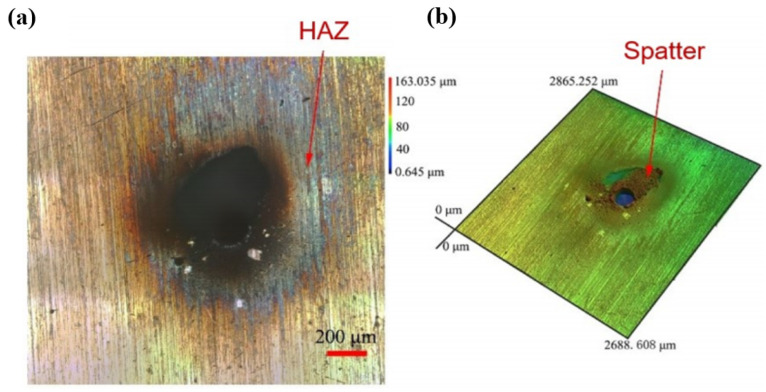
Thermal effect of femtosecond laser through-hole drilling at repetition rate of 500 kHz. (**a**) The actual condition of the hole surface. (**b**) Three-dimensional simulation of the hole surface [57].

Xia et al. [57] studied the morphology and roughness of a through-hole sidewall, which was processed using a femtosecond laser on stainless steel 304 (SS304). As can be seen in Figure 4a, a distinct thermal effect occurred near the through-hole entrance when the pulse repetition rate was 500 kHz, which included an HAZ and an oxidation zone. In addition, there was some spatter near the through-hole entrance, which was due to the thermal effect. At higher pulse repetition rates, the continuous action of high repetition-frequency femtosecond laser pulses led to heat accumulation in the material, resulting in conductive heating and heating effects. Figure 4b depicts the 3D simulation of the hole surface. Moreover, the composition of the material at a point in the oxidation zone near the through-hole entrance could be determined using energy-dispersive spectrometry (EDS).

**Figure 5:**
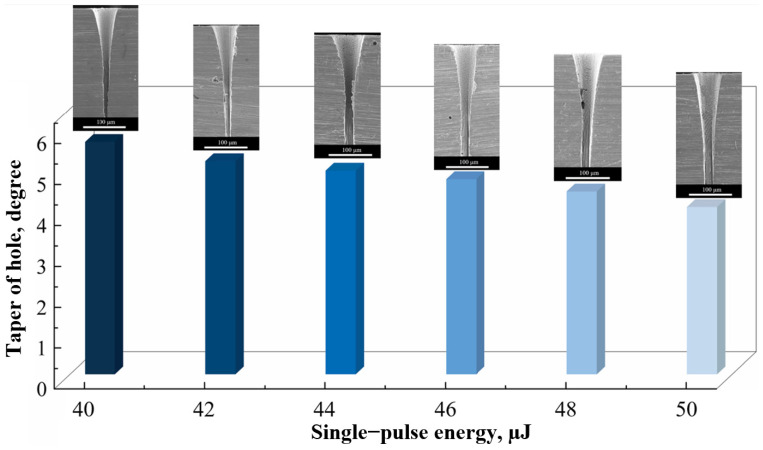
Taper of the hole and the corresponding cross-sectional morphology under different second stage energies [58].

Zhang et al. [58] analyzed the cluster evolution in a simulation system for the femtosecond laser irradiation of Ni. Based on molecular dynamics, they also proposed a two-stage pulse energy (TSPE) percussion drilling process. In this process, in the pulse sequence, the second-stage energy is higher than the initial-stage energy. Figure 5 shows the taper of the hole and the corresponding cross-sectional morphology under different second stage energies. It is clearly shown that a minimal taper was obtained when the second stage pulse energy was maximal, and an initial energy of ~40 μJ and a second stage pulse energy of ~50 μJ provided the optimal combination for TSPE percussion.

**Figure 6:**
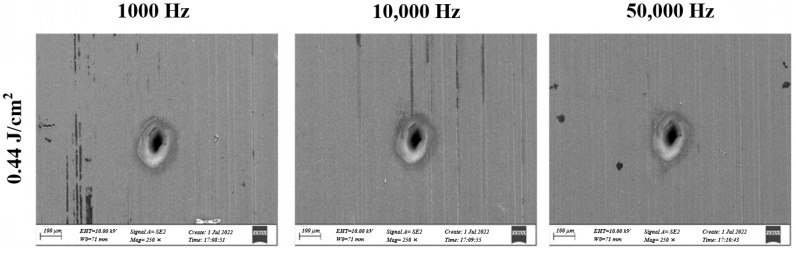
SEM image at different laser fluence and pulse repetition rate of 0.44 J/cm^2^ for 200 µm hole diameter [59].

There are a few studies on the effects of laser drilling parameters on the pore morphology of titanium alloys using solid-state laser drilling technology. Deepu P. et al. [59] reported the ablated micro hole features obtained when using a femtosecond laser on Ti6Al4V. At a fluence of 0.44 J/cm^2^, a repetition rate of ~10 kHz, and a pulse overlap of ~85%, a better hole shape could be obtained, without considering the hole size or laser fluence, as can be seen in Figure 6. Furthermore, at a higher pulse overlap, micro-cracks, HAZs, and recast layers were more prominent, which was due to the higher heat accumulation, as titanium alloy has a lower thermal conductivity.

**Figure 7:**
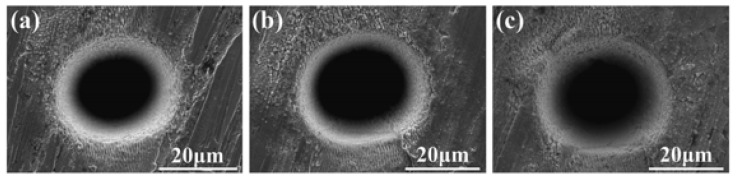
Surface morphologies of holes under different numbers of pulses: (**a**) 1 × 10^6^ pulses; (**b**) 2 × 10^6^ pulses; (**c**) 4 × 10^6^ pulses [60].

Zhang et al. [60] conducted multi-pulse femtosecond laser micro-drilling tests on refractory tungsten (W), and they compared the micro-drilling quality and hole-forming mechanism of W under different conditions. The profile and morphology of holes are significant indicators of their quality, after processing using femtosecond laser drilling. When the numbers of pulses were 1 × 10^6^, 2 × 10^6^, and 4 × 10^6^, the hole mouths were round, with smooth hole walls and no obvious HAZs; the surface morphologies of the holes are shown in Figure 7. In Figure 7c, it can be clearly seen that only a few high-quality particles were attached to the sample surface. Furthermore, the authors also stated that significant thermal accumulation did not lead to grain growth during the femtosecond laser ablation processing of W.

Over all, ultrashort laser micromachining is preferred over long-pulse laser micromachining in several unconventional processes, which is due to its ability to achieve high-precision processing and a wide processing range. Therefore, ultrashort-pulse lasers overcome the disadvantages of long-pulse lasers in the processing of difficult-to-cut materials, ultrahard materials, and complex geometry materials. Table 1 presents the characteristic information of metal material drilling with femtosecond lasers.

### 3.2. Metal Ablation Thresholds Using Femtosecond Lasers

Unlike traditional tools, lasers, as a new type of processing tool, do not use sharp blades for cutting and destroying materials. Instead, they use complex physical and chemical processes [61]. Pulsed lasers have the characteristics of a short working time, a high machining accuracy, and they can be used to machine a wide range of materials. They are widely used in various fields, such as the micromachining, medical treatment, and aerospace fields. Femtosecond lasers have a low cost, a high processing efficiency and micron processing accuracy, and are easy to maintain [62]; thus, they have broad application prospects in industrial production. With the continuous development of laser technology, the interaction between pulsed lasers and material surfaces has become a hotspot for researchers.

**Figure 8:**
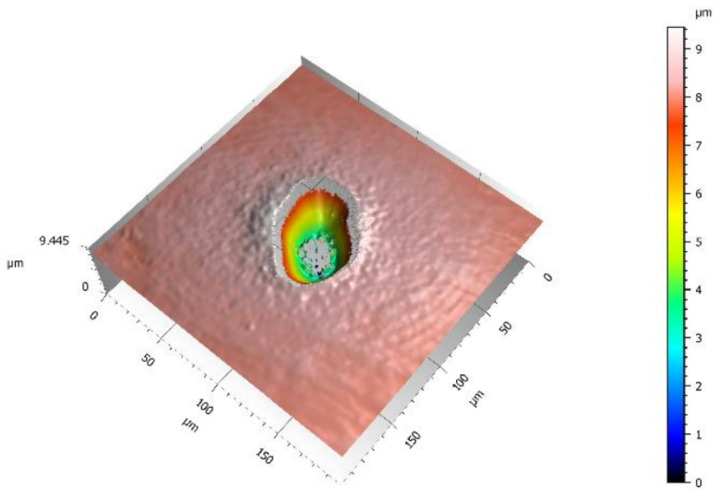
Three-dimensional view and two-dimensional profile of Ti-6Al-4V surface ablated with a pulse energy of 19.5 µJ and 100 shots [64].

The ablation threshold represents the minimum energy density required for material removal using lasers [63]. The main requirement for laser removal is that the energy density of the laser is greater than the ablation threshold. The ablation threshold is an inherent parameter related to the laser itself and the material properties, and it is usually determined in experiments. Maharjan et al. [64] proposed that the laser ablation threshold could be determined by either the diameters or depths of laser-induced craters, for which the threshold is more credible than that determined using the former method. Moreover, they also obtained an ablation threshold of ~0.142 + 0.010 J/cm^2^ for 100 laser pulses at a 130 fs pulse duration on Ti-6Al-4V alloy, as shown in Figure 8.

**Figure 9:**
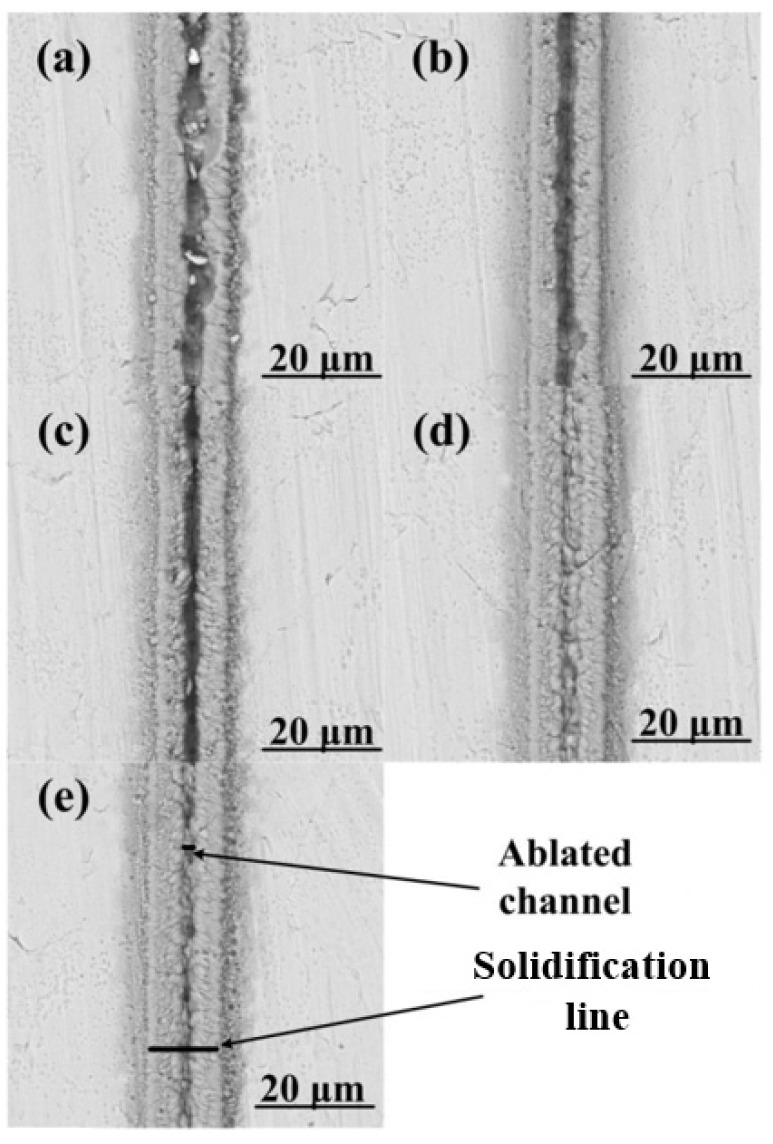
SEM images of the processed regions: (**a**) 100 μm/s; (**b**) 200 μm/s; (**c**) 300 μm/s; (**d**) 200 μm/s; (**e**) 500 μm/s [65].

A new ablative channel width prediction model was established by Liang et al. [65], and the ablation threshold of materials that were processed using a femtosecond laser was also calculated. To verify the accuracy of the model, a 50 MHz femtosecond laser with different scanning speeds was used to process an SUS 301 stainless steel sheet, which can be seen in Figure 9. The authors compared the calculated threshold with the threshold determined using the previous method, finding negligible errors.

**Figure 10:**
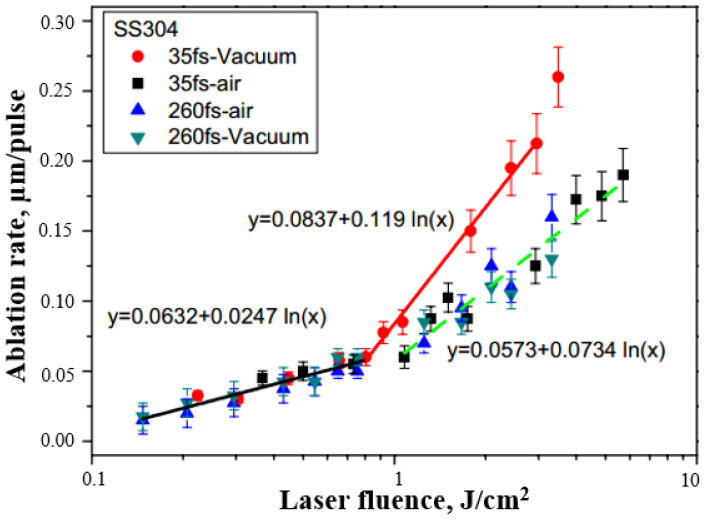
Dependence of ablation rate on laser fluence in air and vacuum (N = 200 pulses) [66].

Xu et al. [66] studied the ablation rate of 304 stainless steel (SS304) as a function of laser pulse fluences in air and under a vacuum. They proposed that the two ablation regimes were dominated by either optical penetration (α-1) or the electron heat diffusion length (L). In the low fluence regime, the single-shot ablation threshold Fth(1) was ≈0.077 ± 0.008 J/cm^2^, while in the second fluence regime, it was 0.46 ± 0.05 J/cm^2^ (35 fs, in air). Furthermore, regardless of the pulse durations and environments, there were no differences in the ablation rates at low laser energies (<0.92 J/cm^2^). However, in the high laser fluence range (>0.92 J/cm^2^), the ablation rate of 35 fs laser pulses in a vacuum was higher than that of the others, as depicted in Figure 10.

Mensink et al. [67] studied fast, high precision machining of difficult-to machine materials through femtosecond laser ablation. In order to solve heat accumulation melting without the shielding effect, a high repetition frequency (57.4 MHz) and an ultra-low femtosecond laser was used to irradiate fine crystal tungsten carbide in order to determine its incubation effect and ablation threshold for subsequent heat accumulation melting. It was found that heat accumulation at high repetition rates explains the ultra-low flux melt threshold behavior that produces melt crowns around ablative holes and grooves. The results of this study are helpful in predicting the heat accumulation effect under high repetition rate laser irradiation and in realizing the molding application of difficult-to-process and superhard materials.

Genieys et al. [68] provided measurements of the ablation for four metals (aluminum, copper, nickel, and tungsten), which were irradiated using single 800 nm laser pulses in a regime lasting from 100 fs to 15 fs. The threshold values obtained for each pulse duration and metals are shown in Table 2. It was clear that, for all the metals tested, the ablation threshold was constant over the investigated duration (15–100 fs). In fact, the small changes observed in different pulse durations were lower than the error, which was closely related to the uncertainty of the threshold determination.

**Figure 11:**
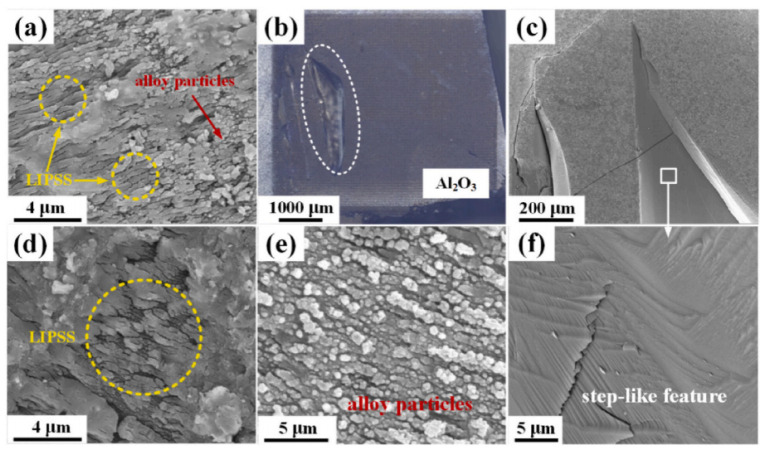
The typical fracture surface morphology of Fe–36Ni/sapphire joints (welded with a laser scanning speed of 50 mm/s) after shear strength tests on the sapphire side [69]. (**a**) the microstructure of the fracture surface occurred at the interface; (**b**) the macroscopic morphology of the fracture surface at Fe–36Ni alloy side; (**c**) the microstructure of the fracture surface located in the sapphire substrate close to the interface; (**d**) the magnified image of the marked zone II in (**a**); (**e**) the magnified image of the marked zone I in (**a**); (**f**) the magnified image of the marked zone in (**c**).

Using femtosecond laser micro-welding technology, Pan et al. [69] successfully realized the direct joining of sapphire with Fe-36Ni alloy for the first time. An excellent joint without holes and micro-cracks was obtained, with an interface width of less than 1 μm. No obvious element diffusion or metallurgical reaction occurred at the interface. The authors proposed that, for a particular material, when the laser irradiation intensity is slightly higher than the ablation threshold, the laser-induced periodic surface structure (LIPSS) can only be formed by laser irradiation; if the laser irradiation intensity was at a higher laser fluence, it would produce random micro- and nano- particles or periodic micro structures. Figure 11 shows the typical fracture surface morphology of Fe–36Ni/sapphire joints (welded with a laser scanning speed of 50 mm/s) after performing shear strength tests on the sapphire side.

The ablation properties depend largely on the physical and chemical properties of the material. The effect of these factors on the ablation rate is described by the ablation threshold, which is the minimum energy required to initiate the ablation of the material. When increasing the number of incident laser pulses, the ablation threshold tends to decrease, following the exponential power law of the inoculation factor. For metals, the behavior of this ablation threshold is interpreted as leading to a laser-induced plastic deformation of the thermal stress field. Overall, the basic physical mechanisms of material removal during laser ablation are not fully understood, and, therefore, researchers are still currently investigating multiple methods that can be used to characterize and optimize the process. The metal ablation thresholds using femtosecond lasers are summarized in Table 3.

### 3.3. Micro/Nano-Surface Modification Using Femtosecond Lasers

The laser processing of metal surfaces has significant advantages, such as no-contact treatment, no accelerated fading, digital control, scratch resistance, and environmental friendliness [70,71,72]. Compared with traditional laser processing, femtosecond laser processing, with its benefits of a short laser duration and an accelerated modification process (accelerated before obvious thermal diffusion), is generally considered to be non-thermal. Therefore, nanoscale structures can be easily created on the sample surface, which has gradually become a popular choice for micro/nano-surface modification in recent years.

**Figure 12:**
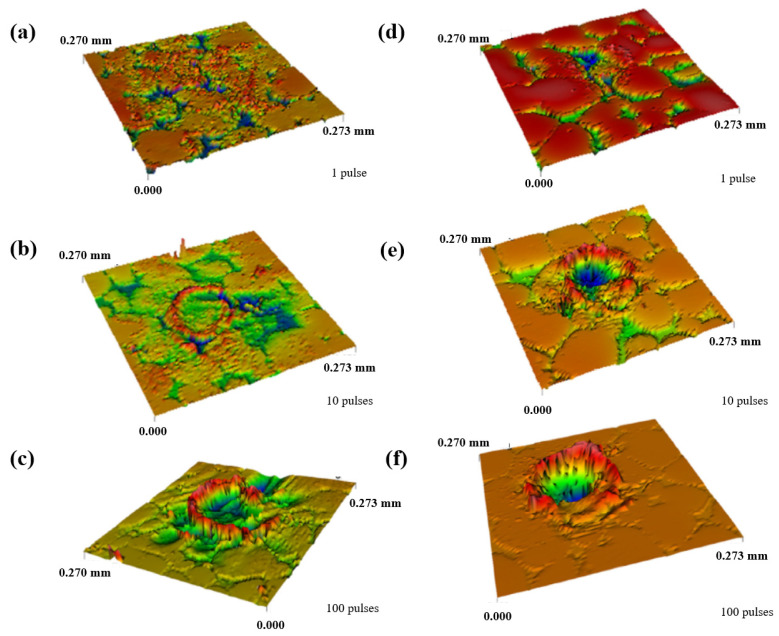
The 3D view of the damages and their cross-section after the laser irradiation of tungsten in air (**a**–**c**) and a vacuum atmosphere (**d**–**f**) with different pulse durations (1, 10, and 100) [73].

Trtica et al. [73] explored the high-intensity femtosecond laser-assisted surface modification of tungsten samples in air and under a vacuum atmosphere. They confirmed that the competition between optical breakdown phenomena and filamentation strongly depends on the geometrical focusing distance. In addition, the ablation thresholds of tungsten targets irradiated using a femtosecond laser under two conditions were also estimated. The threshold fluence decreased as the number of pulses increased, and the thresholds in air and under vacuum showed little difference. Figure 12 depicts a 3D view of the damages and their cross-sections after the laser irradiation of tungsten in air (a–c) and under a vacuum (d–f) with different pulse durations (1, 10, and 100). It was found that the maximum depth of the crater in a vacuum (~31 µm) was higher than that in an air atmosphere (~7.2 µm), which was closely related to the hydrodynamic effects in a vacuum.

**Figure 13:**
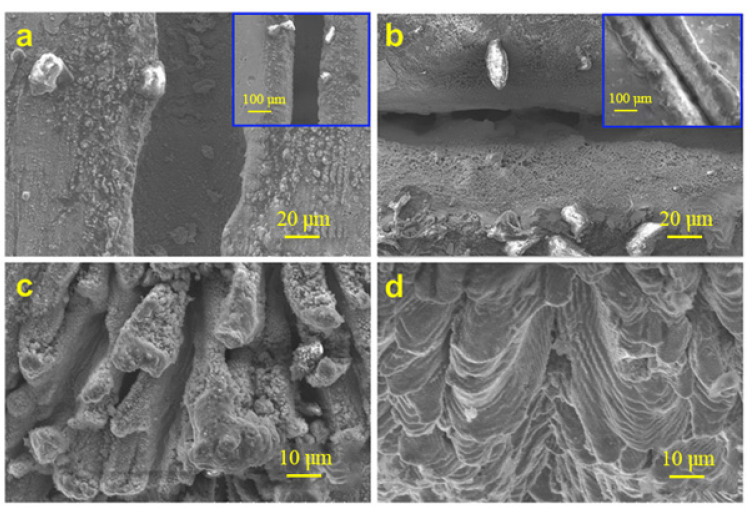
SEM images of (**a**) femtosecond laser trimming and (**b**) nanosecond laser trimming; surface morphology of the Fe-Cr-Al alloys resistor sheet by femtosecond laser trimming (**c**) without and (**d**) with ultrasonication [74].

Su et al. [74] compared the influences between femtosecond and nanosecond laser trimming on Fe-Cr-Al alloys. In their study, femtosecond laser trimming showed higher precision in resistance adjustment than in the nano-direction, which was due to different single-pulse energies, leading to the difference in the quantity of surface etched. Figure 13 presents the scanning electron microscopy (SEM) images of a sample (No. 3921R3). With an increase in pulse width, the temperature of the central electricity slowly changed, and the temperature peak of the lattice increased. The wavelength of the femtosecond laser had an impact on the ablation and optical adsorption properties.

**Figure 14:**
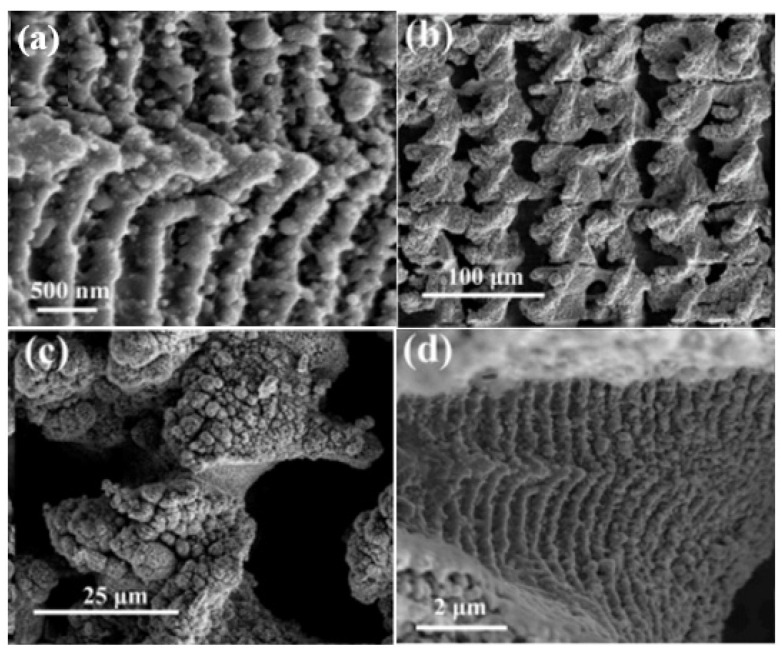
(**a**–**d**) SEM images of the stepwise-magnified micro/nano hybrid structures with optimum femtosecond laser modification [75].

Lou et al. [75] proposed a two-step strategy for constructing antireflective systems on Ti-6Al-4V surfaces through a combination of nanosecond and femtosecond pulsed lasers. Further, they successfully realized ultralow-broadband interface reflection, and the deep air holes that were induced on the surface on the microscale played an important role in this antireflection. The femtosecond-laser-induced nanostructures on the air hole wall further enhanced the multi-reflection process, which led to the satisfactory anti-reflection results. The morphology of the post-treated sample, treated with a femtosecond laser scanning velocity of 12 mm/s, was magnified stepwise, as depicted in Figure 14a–d. This showed that deeper holes enhanced the internal multi-reflection process. The ultra-low reflectance of the hybrid structures was found through the light trapping effect of air holes and the photoimpedance matching effect of the nanostripes.

**Figure 15:**
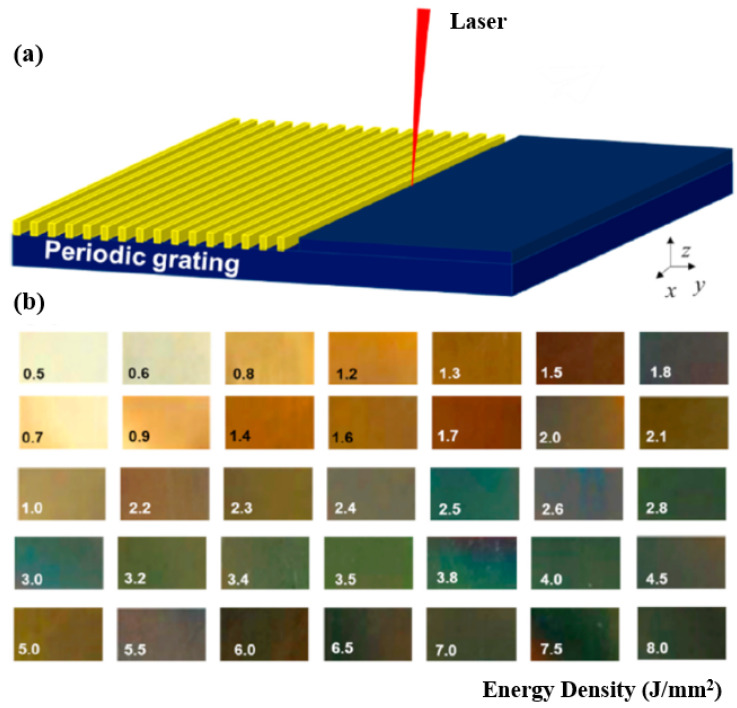
(**a**) Three-dimensional schematic diagram of laser-processing periodic surface grating structure. (**b**) The femtosecond laser processed amorphous alloy surfaces that exhibit various colors with the increase in energy density [76].

Gao et al. [76] reported on the femtosecond laser processing of bulk metallic glass (BMG). However, when the laser energy density increased from 0.5 J/mm^2^ to 8.0 J/mm^2^, the color of Zr-based BMG changed from yellow to brown and then to green, as shown in Figure 15. Their approach provides an easy method for engraving colored amorphous alloy surfaces, thereby broadening the application of BMG.

**Figure 16:**
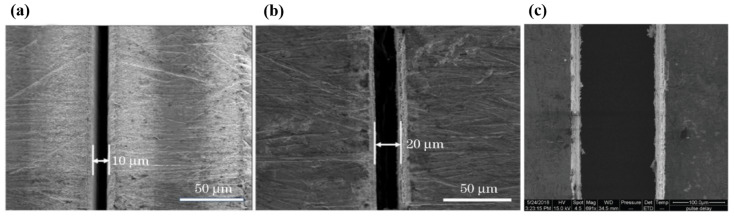
Topographies of microgrooves with widths of 10 (**a**), 20 (**b**), and 150 (**c**) µm [77].

Liang et al. [77] proposed a spatially-shaped femtosecond ultrafast laser-processing system based on the principle of beam shaping, built to modulate a Gaussian beam before focusing on a rectangular flat-top light, and they explored the influence of spatial-shaping light on microgroove structures and reductions in taper. Furthermore, deep grooves with widths of 10, 20, and 150 μm were machined. No obvious HAZs or element changes were found, indicating the great application potential and excellent processing ability of this method for surface modification. Figure 16 shows the topographies of the microgrooves with widths of 10 (a), 20 (b), and 150 (c) µm.

**Figure 17:**
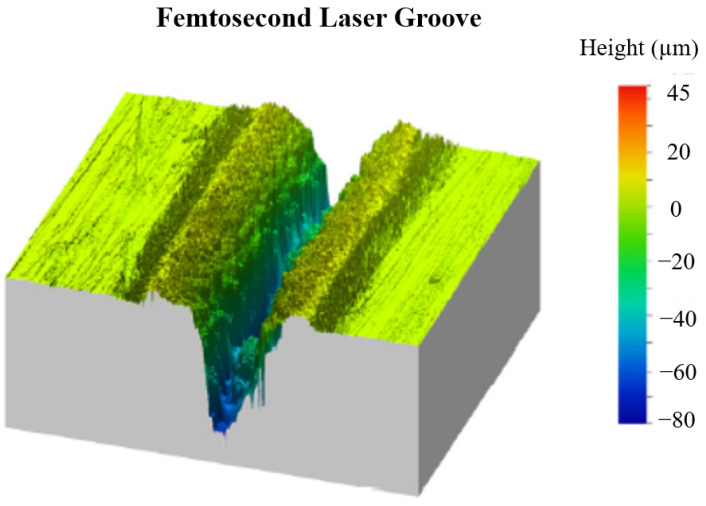
An optical profilometry image of the grooves generated by femtosecond laser [78].

By comparing treatments with nanosecond, picosecond, and femtosecond lasers, Pou-Álvarez et al. [78] explored the effect of the laser pulse length on the surface properties of laser-textured magnesium alloys. In their study, in femtosecond processing, the recast layer was thinner due to the shorter pulse length, which was further reduced during substrate thermalization. It adhered well but with some porosity beside the groove wall and medium-to-low irradiance levels of the Gaussian tails, but this was negligible in the high-irradiance region near the center of the spot. Figure 17 presents an optical profilometry image of the grooves generated by femtosecond laser. It clearly showed that both the decrease in melt displacement and the increase in the length–diameter ratio were clear at shorter pulse lengths.

**Figure 18:**
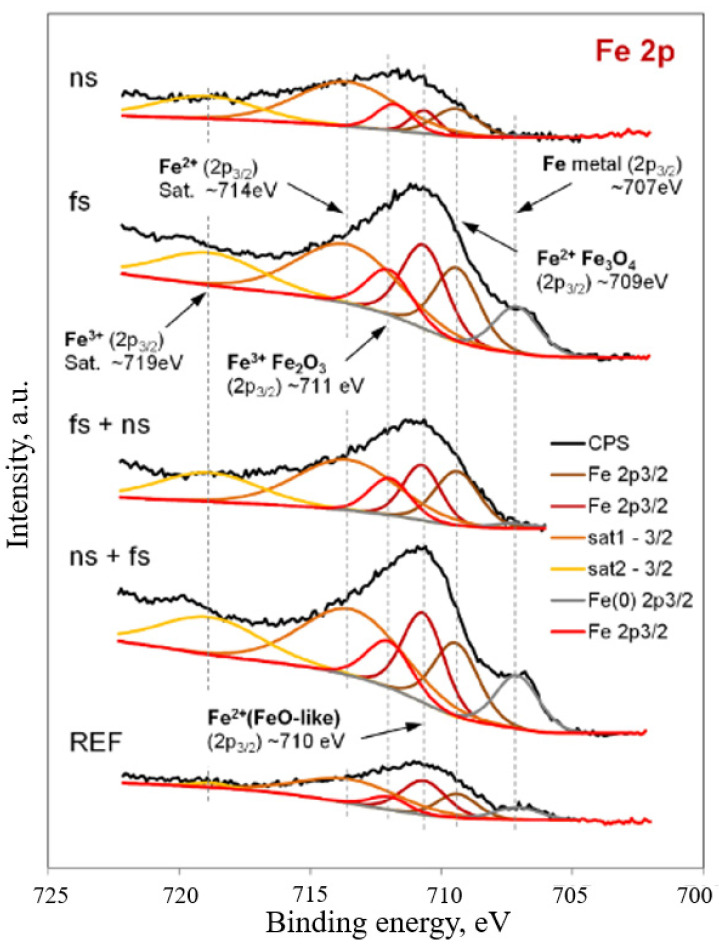
The change in the X-ray photoelectron photoemission spectra lines of the Fe2p [79].

Similarly, Mroczkowska et al. [79] presented an analysis of the irradiation of stainless steel with nanosecond and femtosecond lasers. They concluded that using a femtosecond laser on the early structure of the steel promoted the surface extraction of chromium ions, which contributed to improving the corrosion resistance compared with that of the nanosecond laser-treated samples. However, a more in-depth analysis showed that this also resulted in a higher content of iron ions on the surface. Figure 18 shows the changes in the X-ray photoelectron photoemission spectra lines of the Fe2p.

**Figure 19:**
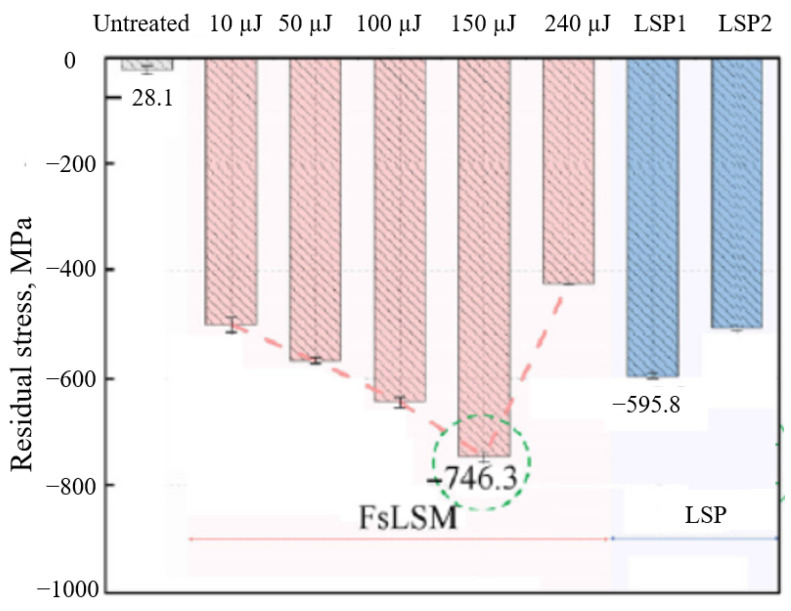
Surface residual stress distribution [80].

Pan et al. [80] proposed a femtosecond laser which was used to irradiate Ti6Al4V titanium alloy surfaces under an air conditioned environment. In their work, the surface microhardness was improved by 16.6%, and the residual stress reached 746 MPa when the laser energy reached 150 µJ; however, when the energy was 240 µJ, a clear drop in residual stress (424 MPa) occurred, which was due to the thermal relaxation of dynamic recovery, as can be seen in Figure 19.

Table 4 summarizes the studies on the micro/nano-surface modification of metal using femtosecond lasers.

Overall, ultrashort pulses mainly have an impact on the micro/nano-surface modification of materials, which is due to the shorter energy deposition time, limiting the production of heat and leading to the occurrence of LIPSSs. Interestingly, these periodic structures are often sub-micron in size, acting as diffraction gratings, and generating structural colors. Laser processes are accompanied by changes in geometry and chemistry, which will also be explored.

### 3.4. Printed Circuit Board Micromachining Using Femtosecond Lasers

The interactions between femtosecond lasers and PCB multi-layer heterogeneous materials are complex [81,82,83]; however, the mechanism of these interactions is still in line with the basic principle that the laser energy is absorbed by the substance, making the atoms or molecules inside the substance eliminate the binding energy, thereby leading to the substance’s ablation. Therefore, the absorption of laser energy by materials and the ability of atoms or molecules to eliminate the binding energy between particles after this absorption are key to the occurrence of ablation.

**Figure 20:**
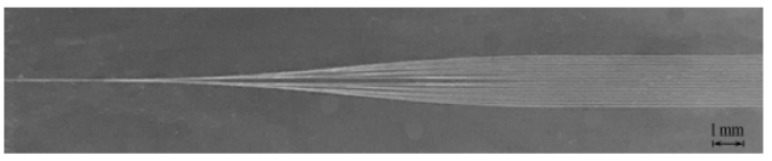
A compact air-cladding 1 × 16 polymeric optical splitter [84].

Tao et al. [84] proposed an improved electro-optical printed circuit board (EOPCB) structure, for which a compact 1 × 16 polymeric optical splitter was fabricated using the beam propagation method, as illustrated in Figure 20. In their study, the average insertion loss per channel for the splitter was IL ≤ 20 dB, and the uniformity was 1.42 dB. Furthermore, they explored the optimal machining parameters (the etching power was 100 mW, etching speed was 10 mm/s, and the groove-gap was 4 µm).

**Figure 21:**
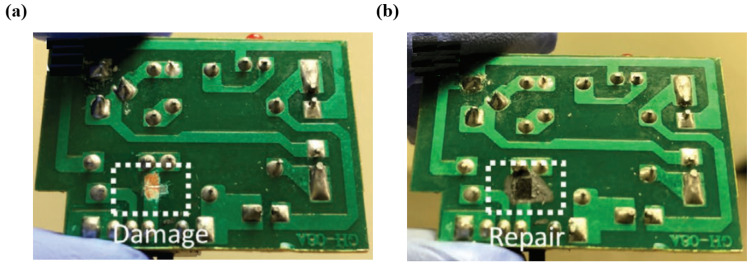
A demonstration for PCB repair [85]. (**a**) Damaged track in the control PCB, (**b**) Repaired track using FsLDW of rGO.

Lim et al. [85] presented an innovative approach for repairing circuits on flexible PCB substrates, utilizing laser printing to create conductive traces through reduced graphene oxide (rGO) and a femtosecond laser direct writing (FsLDW) platform. The utilization of FsLDW enabled the creation of conductive rGO traces, which can be used to print new traces, repair damaged traces, and add additional sub-circuits above the original circuit with a dielectric graphene oxide (GO) layer in between. The adjustability of the trace width, thickness, and sheet resistance of the patterned rGO was evaluated by varying the pulse energy, scanning speed, and repetition rate within the FsLDW structure. Figure 21 demonstrates PCB repair. Deliberate damage was inflicted on the power lines in the PCB by cutting the copper track (Figure 21a). A layer of GO was applied over the affected area, and a novel conductive pathway was created using the proposed FsLDW technique (Figure 21b).

**Figure 22:**
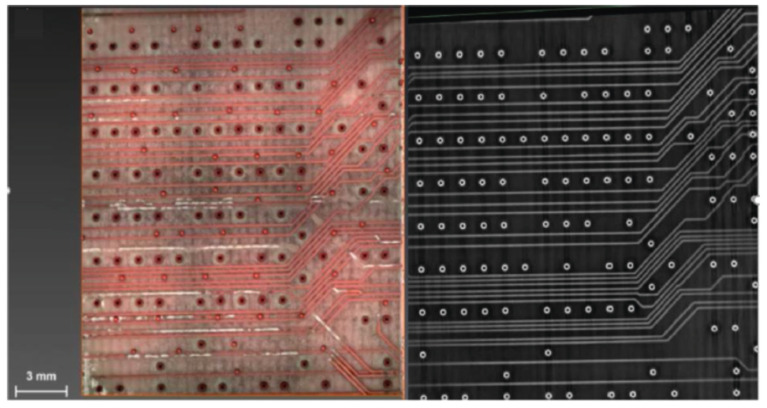
A side-by-side of reconstructed volume and X-ray computed tomography (CT) of PCB [86].

Choi et al. [86] presented a novel workflow that integrated an ultrashort-pulse laser for precise and replicable material removal, along with digital microscope imaging for information acquisition, enabling a fully automated PCB reverse engineering process. They also demonstrated the effectiveness of this approach on a PCB sample and validated their findings through a comparison with X-ray images. Figure 22 presents a side-by-side view of the reconstructed volume and X-ray computed tomography (CT) images of the PCB. The dataset obtained using the proposed method not only includes all the information from the X-ray CT image but also reveals additional details that were not captured by X-ray CT.

**Figure 23:**
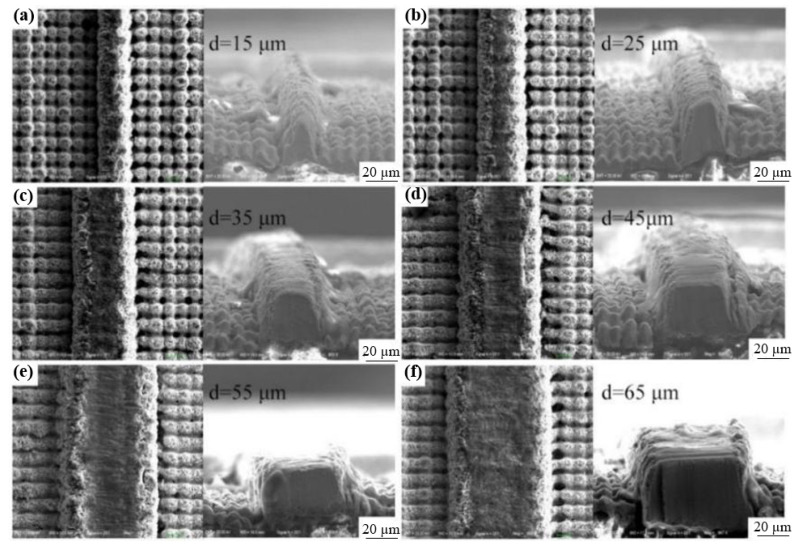
SEM images of copper tracks manufactured on PCB with different the track’s pre-defined width d) [87]. (**a**) d = 15 µm; (**b**) d = 25 µm; (**c**) d = 35 µm; (**d**) d = 45 µm; (**e**) d = 55 µm; (**f**) d = 65 µm.

Lu et al. [87] investigated the effects of the femtosecond pulse frequency and power on the surface roughness, copper content, and micromachining depth of machined zones. They found that, in the condition of a pre-defined track width of ~25 µm, a single conductive line appeared, and two parallel copper tracks were created, with no evidence of copper in the ablation zones; the pre-defined spacing was more than 45 µm. Figure 23 presents SEM images of the copper tracks with different widths micromachined on PCBs; the cross-section was triangular or trapezoidal, rather than an ideal rectangle. Moreover, they also found that an average machining accuracy of ~0.67 µm showed great potential in the field for creating high-density and precise patterns on PCBs.

It can be observed that, in accordance with the requirements of the circuit board manufacturing industry, the utilization of laser processing for circuit boards is a significant application direction within current femtosecond technology. In particular, the process of laser ablation and the direct formation of conductive lines during circuit board fabrication enhance both the manufacturing process and structural optimization. The implementation of femtosecond lasers in fine conductive line formation has proven to be highly beneficial.

Table 5 summarizes the key parameters of PCB micromachining using femtosecond lasers.

### 3.5. Liquid Metal Processing Using Femtosecond Lasers

Flexible electronic devices, which generally use integrated electronic components, are attracting increasing attention due to their wide application potential in biological research, medical treatment, and health monitoring [88,89,90]. Conventionally, metal nanoparticles, metal nanowires, and ionic gels are embedded in elastomers to form conductive elastomers with an excellent conductivity under strain. Recently, due to its desirable characteristics, LM has been applied as an intrinsically flexible electrode material in the fabrication of flexible electronic devices [91]. It shows great potential in health monitoring, medical treatment, soft robotics, etc.

**Figure 24:**
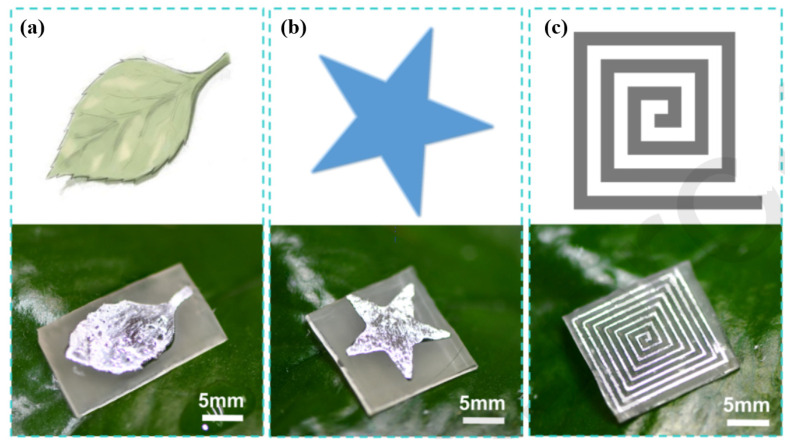
Different shapes of the LM patterns. (**a**) Leaf-like pattern, (**b**) star-like pattern, and (**c**) complex spiral pattern [92].

Professor Feng Chen’s team at Xi’an Jiaotong University has conducted an extensive and in-depth exploration of the application of LM in flexible circuits. In 2020, this team realized a novel way to prepare liquid metal-repellent surfaces with an 800 nm femtosecond laser, exhibiting excellent repellence to Ga-based LM, which was conferred by a polydimethylsiloxane (PDMS) surface [92]. Furthermore, the team performed a simple sandpaper abrasion test to investigate the wear resistance of laser-induced microstructures. When the abrasion time increased, the metal contact angle on the treated PDMS surface was larger than 150°, and the metal sliding angle was below 10°. Strong mechanical robustness and chemical stability were demonstrated on the laser-induced liquid metal-repellent surface. Figure 24 shows the different shapes of the LM patterns.

**Figure 25:**
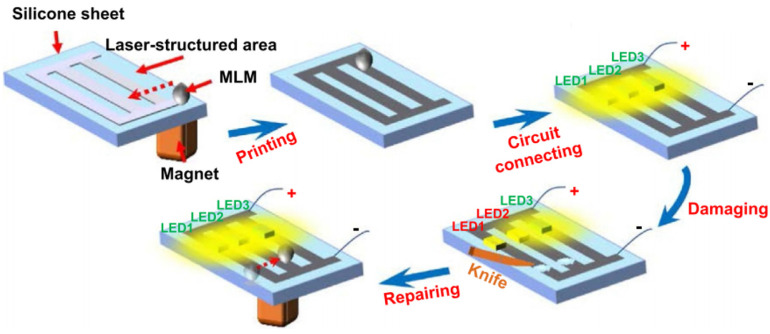
The schematic of repairing a printed LM circuit through a magnetic field-controlled MLM droplet [93].

In 2021, the same team proposed a method for guiding a magnetic liquid metal (MLM) droplet in order to print and repair a flexible LM circuit on an fs laser-patterned silicone surface [93]. The sample surface was illuminated by the fs laser, and the substrate was ablated, forming a silicone surface with supermetalphobicity. The contact angle of the MLM droplet reached 169.5° ± 3.5°, and the MLM droplet rolled away quickly (sliding angle = 5.0° ± 1.0°). Figure 25 presents a schematic of the repairing of a printed LM circuit using a magnetic field-controlled MLM droplet.

**Figure 26:**
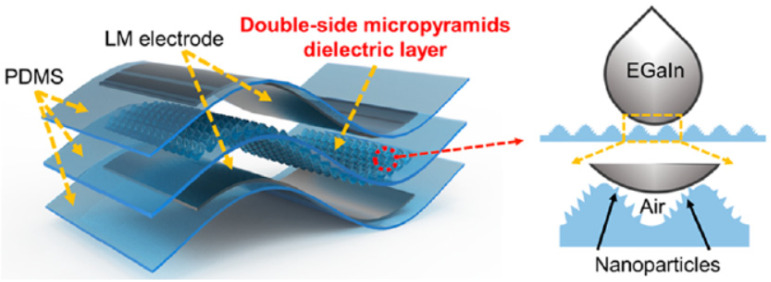
Assembly of the capacitive pressure sensor (**left**) and schematic of the liquid metal on the laser-structured dielectric layer (**right**) [94].

Similarly, in 2022, this team also successfully developed a highly sensitive all-flexible tactile sensor (AFTS) [94], which combines a double-sided micropyramid dielectric layer and an LM electrode using a femtosecond laser fabrication technique, as shown in Figure 26. The sensor exhibited a pressure sensitivity of 2.78 kPa^−1^, an ultralow pressure detection limit of ~3 Pa, a fast response time of 80 ms, and an ultrahigh cycle durability of more than 10,000 times. Due to the femtosecond laser treatment, the external substrate demonstrated an excellent anti-fouling performance and a stable sensing signal in high humidity environments. The successful monitoring of human physiological and motor signals illustrates the potential of this developed AFTS in wearable biomonitoring applications.

Overall, LM combines the advantages of the high electrical and thermal conductivity of metals and the fluid characteristics of water, and it has great potential in the fields of flexible electronics and flexible robots. However, the surface properties of LMs are different from those of simple fluids, which is due to the outside solid oxide layer. For the sake of expanding its applications, it is necessary to study the basic properties of LM, such as infiltration on the surface of materials. As a precision machining method, laser irradiation plays a key role in the preparation of high-performance flexible electronic devices.

Some of the above characteristics of LM processing using femtosecond lasers are summarized in Table 6.

**Figure 27:**
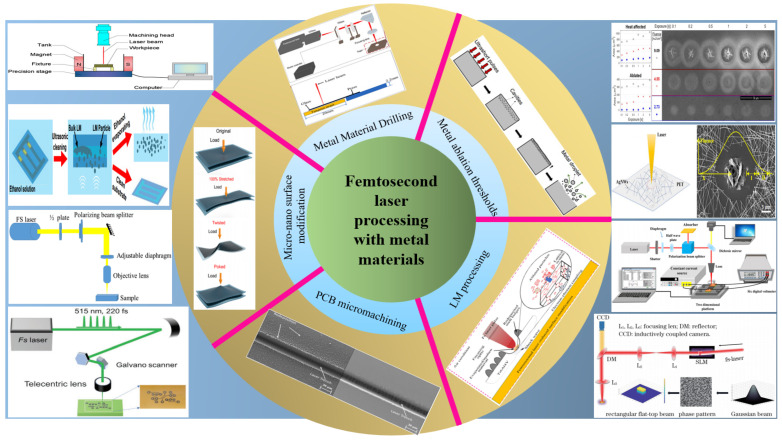
The overview diagram of the classification of metal materials processing by femtosecond laser [57,60,64,65,67,74,75,77,80,86,92,93,94,95].

To date, research on metal material processing using femtosecond lasers has developed rapidly, and the feature size ranges from the micrometer to the nanometer level. Moreover, due to its selective ablation and cold working characteristics, femtosecond machining has become a powerful method that plays a significant role in promoting the transformation of traditional and new industries. This will lead to dramatic improvements in the performance of the micromanufacturing field. A summary of metal material processing using femtosecond lasers is shown in Figure 27. In addition, femtosecond lasers can also be extensively applied in multi-photon fusion, microfluidic chip fabrication, and other processes, and this has injected vitality into the development of optical micro-devices.

## 4. Challenges, Outlooks, and Conclusions

In general, metal material processing using femtosecond lasers is a hot topic in the field of micro-processing because of the distinctive properties of lasers [96,97,98]. In this paper, we reviewed the principles and applications of metal material processing using femtosecond lasers, including the typical properties of lasers. We also discussed the characteristics of laser processing and the theory of metal material processing. Then, we focused on studies published in the last 5 years regarding the femtosecond laser processing of metal materials, including those on metal material drilling, metal ablation thresholds, micro/nano-surface modification, PCB micromachining and LM processing using femtosecond lasers.

However, due to the complexity of the interaction between femtosecond lasers and metal materials, there are still a large number of unsolved problems that need to be explored in terms of the following aspects:

(1) According to the above analysis, the research on the femtosecond laser processing of metal materials is mainly based on single-pulse femtosecond lasers. However, in actual applications, multi-power or multi-intensity pulse lasers are mostly applied, and the cumulative effect of multi-pulse lasers cannot be ignored; thus, the two-temperature model of the interaction between materials and multi-pulse femtosecond lasers needs to be improved.
(2) Moreover, the femtosecond metal processing technique is based on the two-temperature model, which involves determining the laser’s impact on the electron–lattice energy transfer. The high electron temperature during this process affects various electronic physical parameters, including subsequent quantization correction in heat transfer. By combining the dual-temperature equation with a molecular dynamics simulation, a comprehensive understanding of the energy transfer during ultrafast laser processing may be achieved at both the macroscopic and microscopic levels.
(3) The ultimate goal of theoretical research is to guide practice. At present, femtosecond laser processing is at the laboratory stage, and it has not yet been developed for commercial large-scale applications. Therefore, lots of experimental work is needed to continue to obtain the experimental data and summarize the process parameters that affect processing quality and accuracy so as to realize precision machining and make full use of the processing advantages of femtosecond lasers.

Processing using femtosecond lasers has proven to be a promising technique for the direct joining of materials and to be beneficial for the manufacture of optomechanical components. Additionally, these fabrication methods have expanded the application range of laser microfabrication, laser surgery, and micromachining. Therefore, metal material processing using femtosecond lasers produced by a combination of nonlinear optics and plasmon optics should be considered as a new development direction. This will not only add to the literature on the basic properties of plasmon machining, but will also provide ideas for solving the current problems in related laser research.

## Figures and Tables

**Table 1:** Summary of metal material drilling with femtosecond lasers.

Materials	Laser Equipment	Feature Size	Applications	References	Year
Ni-based and Fe-based superalloys	A wavelength of ~1030 nm, a repetition rate of ~10 kHz, a pulse duration of ~200 fs	Less than 0.1 µm	Extending the application of the traditional two-temperature model to laser fabrication	[55]	2020
Ti alloy and Al alloy	A Ti: sapphire chirped-pulse regenerative amplification laser system (a wavelength of ~800 nm, a pulse duration of ~30 fs, a repetition rate of ~110 Hz) (Wuhan Huaray Precision Laser Co., Ltd., Wuhan, China, HR-Femto-IR-50–40)	20–40 µm	Needing further research on laser micromachining in the field of aerospace, biomedicine, and daily life	[56]	2020
Stainless steel 304	The laser spot diameter was 36 µm, the pulse duration was 276 fs, and the laser wavelength was 1030 nm	Less than 200 µm	Proposing the magnetic field-assisted ultrafast laser drilling technology	[57]	2021
Ni-based superalloy	A fs-laser system generating 350 fs pulses at a wavelength of 1030 nm	150–250 µm	Improving the quality of percussion laser drilling holes while ensuring high efficiency	[58]	2021
Titanium alloy (Ti6Al4V)	A gaussian-profiled laser energy distribution with a wavelength of 1035 nm, average power of 40 W, and a pulse width of 350 fs (Wuhan Huaray Precision Laser Co., Ltd., Wuhan, China, HR-Femto-IR-50–40)	200–500 µm	Exploring the influence of laser fluence, repetition rate, and pulse overlap on hole dimension and morphology	[59]	2023
Tungsten (W)	A PHARO sapphire femtosecond laser, wavelength of ~1030 nm, a maximum frequency of ~1 MHz, a pulse width of ~230 fs	5–40 µm	Providing a guidance for femtosecond laser ablation of refractory materials	[60]	2024

**Table 2:** Ablation threshold fluence of the four metals, for a 15, 30, 50, and 100 fs pulse duration deduced from the diameter regression technique. F_th_ was expressed as the laser peak fluence with the measured incident energy.

Pulse Duration	15 fs	30 fs	50 fs	100 fs
Aluminum, F_th_ (J/cm^2^)	0.232	0.239	0.240	0.229
Copper, F_th_ (J/cm^2^)	0.636	0.651	0.637	0.659
Nickel, F_th_ (J/cm^2^)	0.328	0.331	0.329	0.316
Tungsten, F_th_ (J/cm^2^)	0.521	0.541	0.530	0.531

**Table 3:** Summary of metal material drilling with femtosecond laser.

Materials	Laser Equipment	Feature Size	Applications	References	Year
Ti6Al4V alloy	Titanium: sapphire laser (Quantronix Integra C1.0), a wavelength of ~790 nm, a repetition rate of ~1 kHz	25–30 µm	Emerging as a credible alternative to conventional chemical-based processes in surface cleaning	[64]	2018
SUS 301 stainless steel sheet	The repetition rate of the femtosecond laser is 50 MHz at a wavelength of λ = 1040 nm	2–15 µm	Widely used in laser microfabrication, laser surgery, and biomedical applications	[65]	2018
304 stainless steel	Ti: sapphire A commercial chirped-pulse-amplification Ti: sapphire laser system (Spectra physics), a pulse duration of ~35 fs, a wavelength of ~800 nm	20–41 µm	Showing great importance for texturing anti-reflectivity, color marking, anti-corrosion, and super-hydrophobic surfaces	[66]	2018
Tungsten carbide	Ti: sapphire-based system delivering 230 fs pulses at a central wavelength of 1028 nm (Amplitude Systems MIKAN Ytterbium doped)	1000 µm	Achieving high ablation rates of difficult-to-machine, ultrahard materials and helping to enable the shaping of binderless tungsten carbide	[67]	2019
Four metals (aluminum, copper, nickel, and tungsten)	Beam line delivers linearly polarized ~30 fs (FWHM) pulses at 100 Hz with an 800 nm central wavelength (the beam line 5a of ASUR platform at LP3 laboratory)	Thickness varies between 0.5 and 3.2 mm	Serving as rewarding feedback for femtosecond laser micromachining and the laser damage handling of metallic components	[68]	2020
Sapphire/Fe–36Ni alloy	An 1030 nm wavelength (Light Conversion), 1 kHz~1 MHz pulse repetition rate, and a 40 W maximum average power	-	Appearing as a promising technique for the direct joining of materials, and beneficial for the manufacturing of optomechanical components	[69]	2023

**Table 4:** Summary of the studies on the micro/nano-surface modification of metal using femtosecond lasers.

Materials	Laser Equipment	Feature Size	Applications	References	Year
Tungsten	Ti: sapphire laser (PULSAR, Amplitude Technologies, based on CPA technique), with wavelength of 804 nm, 60 fs pulses with 12 mJ peak energy	10–80 µm	Leading to precise superficial material removal, which implies the possibility of ultra-precise surface processing	[73]	2019
Fe-Cr-Al alloy resistor sheet	35 fs pulses at a 1 kHz repetition rate with the central wavelength at 800 nm	5–10 µm	Demanding a higher machining precision for intelligent manufacturing and automatic controlling	[74]	2020
Ti-6Al-4V (TC4) titanium alloys	The femtosecond laser source (Pharos, Light Conversion, Lithuania) had a wavelength of ~1030 nm, pulse duration of ~800 fs, repetition rate of ~100 kHz	20–100 µm	Having multiple functions in metal surface modification	[75]	2020
Zr-based amorphous alloy	Linearly polarized femtosecond laser (TCR-1060), wavelength of ~1030 nm, repetition rate of ~500 kHz, pulse width of ~500 fs	0.05–0.25 µm	Providing a facile method for engraving colorful amorphous alloy surfaces	[76]	2021
Nickel-based superalloy	Wavelength of ~800 nm, a repetition rate of ~1 kHz, pulse width of ~50 fs	5–150 µm	Becoming an essential processing method, especially for difficult-to-process materials	[77]	2021
AZ31B magnesium alloy	A femtosecond Yb: KYW laser source (Spectra Physic) with wavelength of ~520 nm, frequency of ~200 kHz, pulse length of ~2.7 × 10^−15^ s	1–20 µm	Modifying the surface of magnesium, aiming at the improvement of the corrosion properties as well as the generation of a controlled roughness	[78]	2021
AISI 304 stainless steel	Trumpf TruMicro Series 2020 fiber laser (Schramberg, Germany) with pulse duration of ~260 fs to 20 ps, wavelength of ~1030 nm	50 µm	Comparing the effect of the laser treatment of AISI 304 stainless steel	[79]	2021
Ti6Al4V titanium alloys	Pulse duration of 290 fs, which was generated from a high power and energy femtosecond laser (Light conversion, Lithuania)	40–300 µm	Offering unique pathways to enhance the tribological performance of materials	[80]	2022

**Table 5:** Summary of PCB micromachining using femtosecond lasers.

Materials	Laser Equipment	Applications	References	Year
Improved EOPCB	A femtosecond laser of 100 fs, peak power of ~5 W, center wavelength of ~800 nm	Increasing the 5G communication rate, and meeting the high integration requirements for the flexible EOPCB	[84]	2020
rGO on flexible PCB substrates	A Yb-doped femtosecond fiber laser (Satsuma HP, Amplitude Systems, Pessac, France), 515 nm wavelength, pulse duration of ~220 fs	Opening new perspectives for real-time PCB repair and wearable electronics	[85]	2021
PCBs	The laser confocal height sensor for measuring height of the sample to ensure accurate laser focus plane (Keyence CL-P070G),with resolution of ~0.025 µm	Offering a universal solution for rapid automated reverse engineering of microelectronic devices	[86]	2022
PCB (three layers: copper layer, polyimide substrate, and adhesive layer)	Ti: sapphire laser (Phoras 15-1000-PP, LIGHT CONVERSION), wavelength of 342 nm, pulse width of 330 fs	Creating high-density and precision patterns on PCBs	[87]	2022

**Table 6:** Summary of LM processing using femtosecond laser.

Materials	Laser Equipment	Applications	References	Year
Liquid Ga-based metal alloys and PDMS	Ti: sapphire laser system (Coherent, Inc., Librausp 1K-he200, Saxonburg, PA, USA), with a pulse duration of 50 fs, central wavelength of 800 nm, and repetition frequency of 1 kHz	Improving machining precision and miniaturizing devices; it has more significant applications in liquid metal printing, microfluidics, soft robots, and wearable devices.	[92]	2020
Liquid metal with some Fe particles		Allowing for more flexible and functional LM manipulation, which shows great significance for exploring soft circuits.	[93]	2021
Gallium-based liquid metal		Monitoring various human physiological and motion signals, depicting the potential for wearable biomonitoring, human–machine interfaces, and soft robotic systems.	[94]	2022

## Data Availability

No new data were created or analyzed in this study. Data sharing is not applicable to this article.
